# Bacterial Programmed Cell Death and Multicellular Behavior in Bacteria

**DOI:** 10.1371/journal.pgen.0020135

**Published:** 2006-10-27

**Authors:** Hanna Engelberg-Kulka, Shahar Amitai, Ilana Kolodkin-Gal, Ronen Hazan

**Affiliations:** Baylor College of Medicine, United States of America

## Abstract

Traditionally, programmed cell death (PCD) is associated with eukaryotic multicellular organisms. However, recently, PCD systems have also been observed in bacteria. Here we review recent research on two kinds of genetic programs that promote bacterial cell death. The first is mediated by *mazEF,* a toxin–antitoxin module found in the chromosomes of many kinds of bacteria, and mainly studied in Escherichia coli. The second program is found in *Bacillus subtilis,* in which the *skf* and *sdp* operons mediate the death of a subpopulation of sporulating bacterial cells. We relate these two bacterial PCD systems to the ways in which bacterial populations resemble multicellular organisms.

## Introduction

“Programmed cell death” (PCD) refers to any form of cell death mediated by an intracellular death program. PCD is classically known as apoptosis [1], a term that originally defined the morphological changes that characterize cell death. Over the last decade, apoptosis was elaborated in metazoans [reviewed in [2–4]) and is more specifically defined as a pathway that permits animals to eliminate damaged or excess cells efficiently while preserving metabolic resources and avoiding an immune response. However, apoptosis is not the only cell-death program. For example, plants both maintain cell number homeostasis and initiate cell death as part of a wound response with no constituents of the metazoan apoptotic program [5]. Also, metazoans eliminate damaged or excess cells through necrosis and autophagy, both programmed genetically [6–8]. The consequences of these forms of cell death differ importantly from those of apoptosis [6–8]. PCD has also been observed in unicellular eukaryotes [9–12] and even in bacteria [13–21].

Here we focus on two genetic programs that initiate cell death in bacterial cultures. The first is mediated by the toxin–antitoxin module *(mazEF)* located in many bacterial chromosomes and mainly studied in Escherichia coli. The second causes death of a subpopulation in sporulating cultures of the bacterium Bacillus subtilis. These systems suggest a multicellular-like character of bacterial populations, an important emerging concept in microbial research.

## Toxin–Antitoxin Modules in Bacteria

One of the best-studied forms of death in bacteria is mediated through specific genetic modules called “addiction modules” or toxin–antitoxin systems. Each consists of a pair of genes that specify two components: a stable toxin and an unstable antitoxin that interferes with the lethal action of the toxin. Found first in E. coli on low copy number plasmids, they are responsible for what is called the postsegregational killing effect. When bacteria lose these plasmid(s) (or other extrachromosomal elements), the cured cells are selectively killed because the unstable antitoxin is degraded faster than is the more stable toxin (reviewed in [14,16,20,22–25]). The cells are “addicted” to the short-lived product, because its de novo synthesis is essential for cell survival. Thus, “addiction modules” were implicated in maintaining the stability of extrachromosomal elements.

Toxin–antitoxin systems, some of which are homologous to these extrachromosomal “addiction modules,” also occur in the chromosomes of many bacteria [26–29]. E. coli has several pairs of such genes, including *mazEF* [13,30–32], *chpBIK* [31,33], *relBE* [34–36]*, yefM-yoeB* [37–39], *dinJ-yafQ* [16], and *ecnA-ecnB* [24]. The most-studied among these, *mazEF* [30,31], is regulatable and responsible for bacterial PCD [13]. Another toxin–antitoxin module that has been studied extensively is *relBE* (reviewed in [24]). Both *relBE* and *mazEF* have the typical toxin–antitoxin genetic organization with an unstable antitoxin (reviewed in [24,32]). However, they are nonhomologous and differ in their structure and mode of action. Their differences were recently reviewed elsewhere [32]. Here we focus on the *mazEF* system and on its relation to programmed cell death.

## *E. coli mazEF* Is a Stress-Induced “Suicide Module” That Triggers Cell Death

The genetic module *mazEF* consists of two adjacent genes, *mazE* and *mazF,* located downstream from the *relA* gene [30,31]. *mazF* encodes a stable toxin, MazF, while *mazE* encodes a labile antitoxin, MazE, degraded in vivo by the ATP-dependent ClpPA serine protease [13]. MazE and MazF interact [13,40]. MazE and MazF are coexpressed and *mazEF* is negatively autoregulated at the level of transcription by the combined action of both MazE and MazF proteins on the *mazEF* promoter P_2_ [41]. Unlike extrachromosomal toxin–antitoxin systems, which are triggered through the loss of the plasmid from the bacterial cell, this system is activated by several stressful conditions that prevent the expression of *mazEF,* and thereby MazE synthesis, and thus trigger cell death. These conditions include: i) extreme amino acid starvation leading to the production of the starvation-signaling molecule ppGpp [13,42]; ii) inhibition of transcription and/or translation by antibiotics including rifampicin, chloramphenicol, and spectinomycin [43]; iii) Doc protein, a general inhibitor of translation which is the toxic product of the “addiction module” *phd-doc* of the plasmid prophage P1. The postsegregational killing effect of P1 *phd-doc* requires the presence of the *E. coli mazEF* system [44]; iv) DNA damage caused by thymine starvation [45] as well as by mitomycin C, nalidixic acid, and UV irradiation [46]; and v) oxidative stress (H_2_O_2_) [46]. Most of the antibiotics and stressful conditions that were used in these studies are well-known to induce bacterial cell death [47–50]. Thus, bacterial cell death results from stressful conditions that trigger the action of the *mazEF* module.

The effect on PCD of thymine starvation deserves special attention. In 1954, Cohen and Barner discovered that a thymine auxotrophic mutant *(thyA)* of E. coli undergoes cell death in response to thymine starvation [51]. This phenomenon, called thymine-less death (TLD), is observed widely in prokaryotes and eukaryotes (reviewed in [48]). Generally, starvation of bacteria for other growth factors is bacteriostatic; TLD uniquely kills. The molecular mechanism of TLD was not understood in any organism until recently with the discovery that in E. coli thymine starvation triggers *mazEF*-mediated cell death [45]. This old enigma is now understood in *E.coli* as follows. Thymine starvation provokes DNA damage involving a unique breaking/twisting of the chromosome into a configuration that defies all the repair/protective mechanisms [48, 52]. Such serious DNA damage reduces transcription from the *mazEF* P_2_ promoter drastically [45]. This should activate the death program [45].

**Figure 1:**
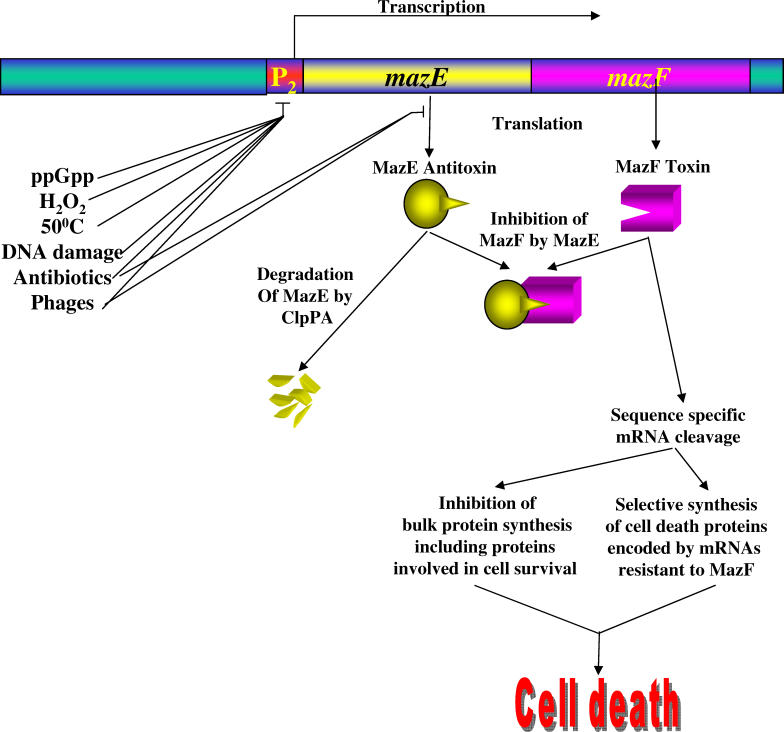
A Schematic Representation of *E. coli mazEF*-Mediated Cell Death For details, see the text.

Thus, *E. coli mazEF* is a stress-induced “suicide module” that activates when a stressful condition interrupts the expression of MazE by preventing its transcription and/or its translation. This leaves MazF unimpeded to exert its toxic effect and cause cell death (Figure 1).

## The Induction of *E. coli mazF*-Mediated Cell Death: A Point of No Return

**Figure 2:**
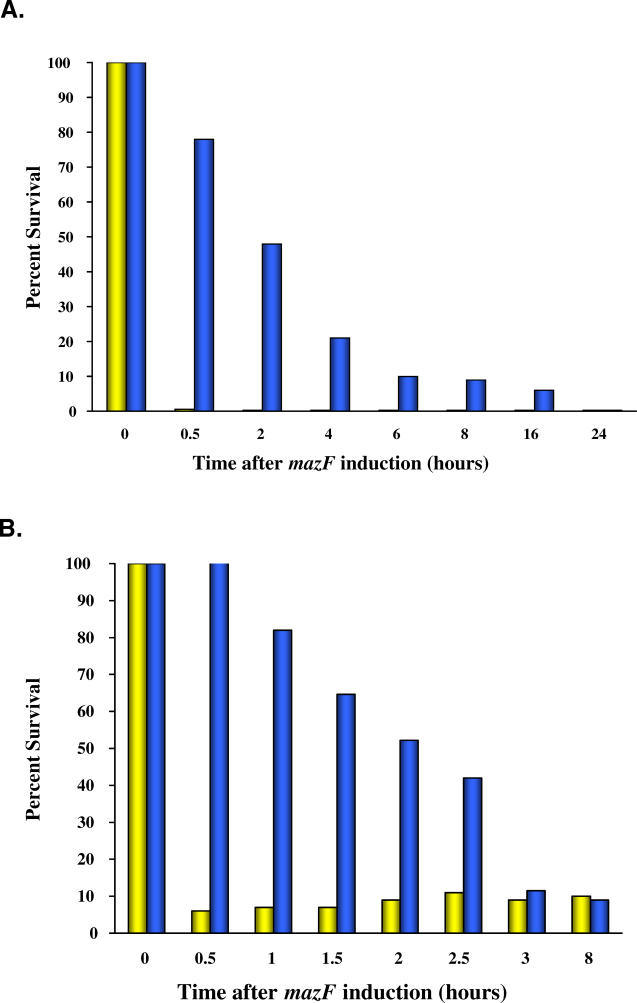
A “Point of No Return” in E. coli after the Induction of the (A) Plasmid Borne or (B) Chromosomally Borne *E. coli mazF* Gene (A) E. coli cells growing in minimal medium were cotransformed with two plasmids, one carrying *mazE* and the other one carrying *mazF,* each regulated by different promoters: *mazF* can be induced by arabinose and repressed by glucose, and *mazE* can be induced by IPTG. At time zero, *mazF* expression was induced by the addition of arabinose. Samples of the induced culture were withdrawn at various time points and spread on plates containing glucose and IPTG (shown in blue on the graph) or glucose without IPTG (shown in yellow on the graph). Based on data in [54]. (B) E. coli cells growing in minimal medium were transformed with a single plasmid-carrying *mazE* that can be induced by IPTG. At the logarithmic phase, stress was induced by the addition of a transcription inhibitor. After the cells were incubated at 37 ^°^C for a short period of time, the transcription inhibitor was removed. Samples of the induced culture were withdrawn at various time points and spread on plates containing IPTG (shown in blue on the graph) or without IPTG (shown in yellow on the graph). The percentage of survivors was calculated by comparing the number of colony forming units (CFUs) of the MazF-induced culture to the number of CFUs of the uninduced culture at time zero. Based on data in [55].

Some debate has surrounded the idea that toxin–antitoxin systems cause PCD rather than bacteriostatic effects. Gerdes and colleagues reported [53] that the toxic effect obtained by an ectopic overproduction of MazF can be reversed by the action of the antitoxin MazE ectopically overexpressed at a later time, and suggested that rather than inducing cell death, *mazF* induces a state of reversible bacteriostasis [53]. However, using a similar ectopic overexpression system, we then found that overexpression of MazE can reverse MazF lethality only over a short window of time (Figure 2A) [54]. There is a “point of no return” which was further confirmed for conditions that are designed to mimic the physiological one [55]; the *mazEF* module was located in its natural context on the E. coli chromosome as a single copy, and *mazEF* was induced by the introduction of one of eight different stressful conditions. In all studied cases, the induction of *mazEF* causes an irreversible loss of viability ([55]; Figure 2B). Thus, a “point of no return,” the basic functional characteristic of cell death, occurs after induction of MazF. That *E. coli mazEF* as well as *relBE* mediate cell death after inducing these modules by stressful conditions (using hydroxyurea) was also shown at the single cell level [56]. These results further support our previous conclusion that *E.coli mazEF* mediates cell death, and it is an active and genetically “programmed” death response. Note that a third gene, *mazG,* whose product, MazG, is a pyrophosphate hydrolase of nucleotides, is located in the *mazEF* operon downstream from *mazF* [57]. Deleting *mazG* decreases cell survival during nutritional stress, and it was suggested that MazG may be involved in restraining cell-death mechanisms so as to delay the point of no return [58].

## How MazF Kills

MazF inhibits protein synthesis through its endoribonucleolytic effect on mRNAs [59–62]. MazF endoribonuclease preferentially cleaves single-stranded mRNAs at ACA sequences [61,62], also tmRNA [59], the tRNA–mRNA hybrids that bind to the A site of ribosomes containing a truncated mRNA, tagging the corresponding nascent polypeptide chains with a degradation signal, while allowing translation to terminate normally (reviewed in [63]). We suggested that the endoribonucleolytic effect of MazF could be one of the initial steps in the programmed cell-death pathway ([2,54,55]. In this model, this initial step can be reversed by the antagonistic effect of MazE over MazF. Further cleavage of mRNAs and tmRNA by MazF would be prevented by MazE, and the previously truncated mRNAs could be released from the ribosomes through the action of de novo synthesized uncleaved tmRNA. However, we suggest [32,54,55] that MazE cannot reverse the downstream cascade already initiated by MazF. Thus, if the process is not stopped in time, eventually cell death would be unavoidable. How might the inhibition of translation by MazF induce such a downstream cascade leading to cell death? Currently, two mechanisms that may act simultaneously seem plausible (Figure 1): i) some of the mRNAs cleaved by MazF encode proteins required for cell survival; and ii) MazF-cleaving mRNAs at specific sites might lead to the selective synthesis of proteins encoded by mRNAs that are resistant to cleavage by MazF. We hypothesize that such MazF-resistant mRNAs might not contain the MazF target site (ACA sequences). Alternatively, they might contain ACA sequences, but could be protected from the action of MazF through some other unknown mechanism. Such proteins could be part of a cell-death network; further work is needed to identify possible pathways to cell death.

## *mazEF* Is Widely Distributed among Bacteria

The *mazEF* toxin–antitoxin system was discovered and studied mainly in *E. coli.* However, *mazEF*-like modules occur in the chromosomes of many other bacteria including pathogens [24,26–29]. The degree of similarity of *mazEF*-like products of various bacteria to that of E. coli is shown in Table 1 with special emphasis on pathogens. Several features are obvious: i) there is no correlation between the presence or the absence of a *mazEF*-like module on the chromosome of a given bacterium and the phylogenetic distance of that bacterium from E. coli. For example, whereas the E. coli close relative Salmonella typhimurium bears no genes similar to E. coli MazE and/or to MazF, gene modules at least 50% similar to E. coli MazE and MazF occur in *Deinococcus radiodurans, Leptospira interrogans, Neisseria meningitidis,* and *Streptococcus mutans,* all of which are phylogenetically remote from E. coli. In *S. mutans,* the *mazEF* module is functional and has a physiological role [64]; ii) sometimes, though, there may be a gene whose product is highly similar to E. coli MazF, the product of the upstream gene is barely similar to the antitoxin MazE. Of course a protein that differs from the E. coli antitoxin MazE might still act as an antitoxin of a *mazF*-like product. For example, B. subtilis has a module called *ydcDE,* of which the toxic product YdcE, called Endo A, is highly similar to E. coli MazF in sequence and structure [65]. Endo A also has an endoribonucleolytic activity, similar to that of MazF, which is inhibited by YdcD. Thus, the antitoxin for Endo A is YdcD, even though it is barely similar to E. coli MazE; iii) the chromosomes of some of bacteria bear more than one *mazEF*-like module.

**Table 1:**
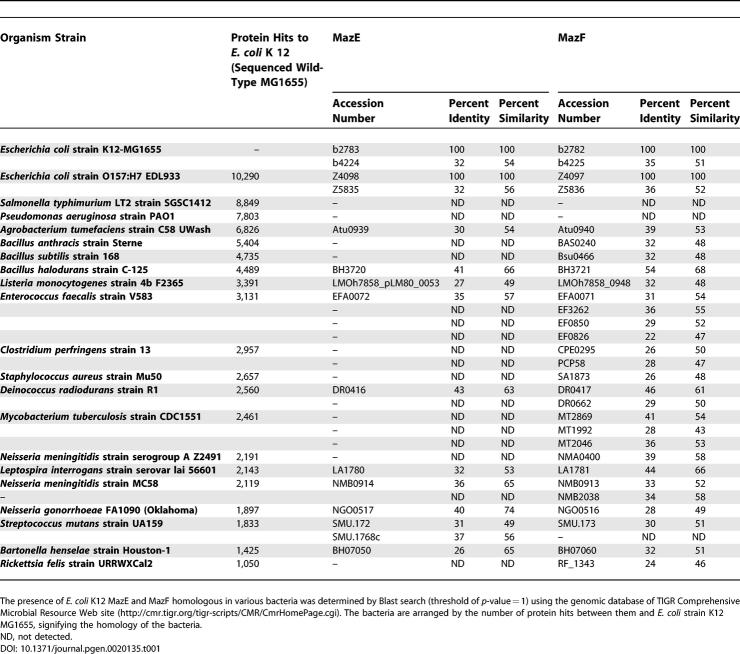
The Distribution of MazE and MazF among Various Bacteria

Mycobacterium tuberculosis is a devastating pathogen in which there may be functional MazF homologs [66]. The chromosome of M. tuberculosis bears at least seven genes encoding MazF-like products (MazF-mt1 to MazF-mt7), some of which (including MazF-mt1) cause cell death when ectopically expressed in E. coli. Purified MazF-mt1 is an endoribonuclease with specificity like that of E. coli MazF [66]. Are these genes expressed and functional in *M. tuberculosis,* or are they only cryptic remainders of some evolutionary process? This question is particularly important because M. tuberculosis chromosome bears no *mazE*-like gene upstream of the *mazF-*like genes.

## Cell Death as a Defense against the Spread of Phage Infection and Other Functions

**Figure 3:**
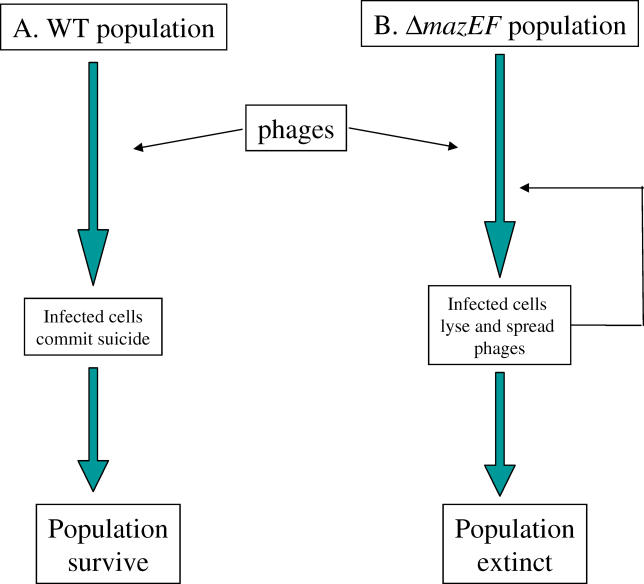
A Model: How Programmed Cell Death Saves a Bacterial Population from Annihilation by Phage P1 Infection (A) In wild-type cells, P1 infection triggers the action of *mazEF* which mediates the death of the infected cells. Because infected cells die before phage can propagate, few phages are released, the titer of the phages is low, and the population survives. (B) In Δ*mazEF* cells, nothing interferes with the phage infections: the infected cells lyse, allowing many released particles to spread to the rest of the population. Thus infection by P1 of a Δ*mazEF* population leads to the death of more cells. Though infection by P1 of wild-type populations leads to the loss of some of the cells, more members of the population survive.

The presence of *mazEF*-like modules in the chromosomes of many bacteria suggests that cell death plays roles in bacterial physiology and/or evolution [28,32]. PCD is clearly counterproductive for an individual bacterium; however, it might be advantageous for a whole cell population. For example, *mazEF*-mediated death can act as a defense mechanism that prevents the spread of phages ([67] and Figure 3). P1 phages exist in two forms: i) virulent particles that are developed in the host cells *(E. coli)* and are released by cell lysis; and ii) as lysogenic prophages that replicate like plasmids using their autonomous origin of replication. Such phages carry a gene coding for a repressor that permits them to replicate in the host cells without entering the lytic phase. Should the repressor become inactivated, prophages enter the lytic stage. Bacterial cells carrying prophages are called lysogens (reviewed in [68]). When Δ*mazEF* P1 lysogens are heat-induced, inactivating a temperature-sensitive repressor, most of the cells lyse, whereas only a small fraction of heat-induced wild-type P1 lysogens lyse [67]. Moreover, the Δ*mazEF* lysogens produce significantly more phages than do the wild-type lysogens. Surprisingly, despite the differences in the level of lysis and phage production, neither wild-type nor Δ*mazEF* cells produce colonies after phage induction. A similar pattern is observed when a virulent phage P1 (which can run only the lytic program) is used to infect Δ*mazEF* or wild-type cells [67]. Why then do neither wild-type nor Δ*mazEF* cells produce colonies after infection? The Δ*mazEF* cells produce no colonies because they are all lysed by the phages. Wild-type cells probably produce no phages because the cells have already been killed by the lethal action of the *mazEF* module. Thus, the *mazEF* module keeps the infection from spreading, thereby protecting the wild-type population from total collapse. The death of individual cells caused by the action of *mazEF* appears to prevent the spread of infective P1 phages. The action of *mazEF* may have a general protective role for the population against a spread of phages [67]. This model, reminiscent of the antiviral response by apoptosis in eukaryotes [69–71], suggests that bacterial populations may share some characteristics of multicellular organisms (discussed below).

Bacterial cell death mediated by *mazEF* may have several other roles [28,32]; the *mazEF* system might act as a guardian of the bacterial chromosome. When, for example, DNA repair systems fail to overcome excessive damage to the chromosome, *mazEF*-mediated cell death might be activated. Thus, by eliminating cells that carry genomic defects and mutations, the *mazEF* system might contribute to the maintenance of genomic stability of the whole population. This potential role for *mazEF* as “guardian of the chromosome” suggests that the presence of *mazEF* on the bacterial chromosome is advantageous to the population because it maintains the continuity of the bacterial population.

Cell death mediated by *mazEF* may also be important in the response of bacteria to severe nutritional stress. When food is scarce, the death of part of the bacterial population may provide nutrients for the surviving cells [13]. This occurs during B. subtilis sporulation, upon the activation of a novel PCD system unlike *mazEF* or other known toxin–antitoxin systems (see below).

## The *B. subtilis skf* and *sdp* Operons Promote Cell Death of a Subpopulation of Sporulating Cells

**Figure 4:**
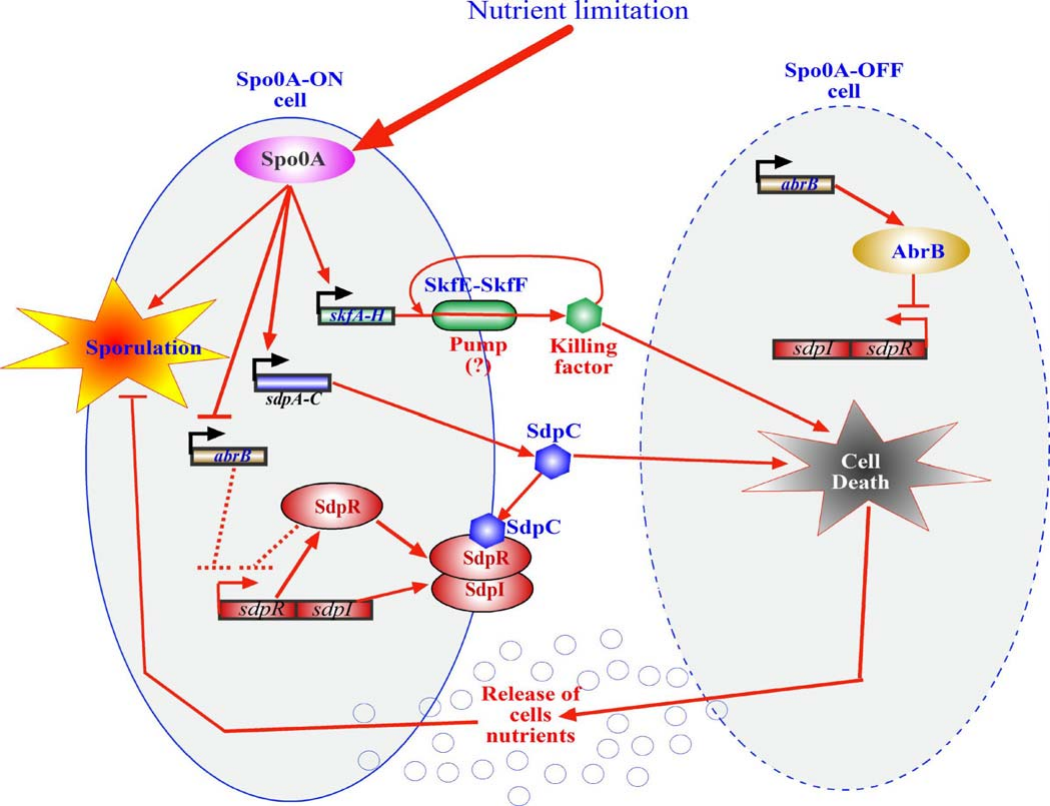
A Model of Delaying Sporulation by Cell Death Nutrient limitation activates Spo0A in a subpopulation (Spo0A-ON cells) of the culture of B. subtilis. Spo0A activates the sporulation process, but can delay sporulation by activating two operons, *skfA-H* and *sdpABC*. *skf* is involved in the production of an extracellular killing factor. SkfE and SkfF, which are produced in Spo0A-ON cells, antagonize the lethal action of the killing factor, probably by acting as an export pump that secretes the factor from the cells. *sdpC* encodes for another killing factor. Two mechanisms are responsible for the resistance of the Spo0A-ON cells to SdpC: i) the three-protein–signaling pathway (SdpC–SdpI–SdpR) (see text); and ii) repression of AbrB synthesis by Spo0A (see text). In Spo0A-OFF cells, the *sdpRI* operon is repressed by AbrB, leading to sensitivity to SdpC toxin. As a whole, Spo0A-OFF cells are killed and lysed, releasing nutrients to be consumed by the Spo0A-ON cells. Thereby, the process of sporulation of the Spo0A-ON cells can be postponed, a potential benefit should food become available [21,74].

Nutrient limitation triggers spore formation in *B. subtilis,* which is governed by the regulatory protein Spo0A [72]. At the inception of sporulation, Spo0A strongly upregulates two operons: *skfA-H* (sporulating killing factor) and *sdpABC* (sporulating delay protein) [73]. Losick and colleagues [21] found that during sporulation the *skf* operon directs the production of an extracellular killing factor (Figure 4). The products of *skfE* and *skfF* confer resistance to the killing factor or toxin. Because SkfE resembles an ATP-binding cassette and SkfF resembles a transport complex (ABC transporter), they might work together as an export pump, exporting the toxin. Produced from the second operon *sdpABC,* SdpC is a 5kDa extracellular factor that acts as an intercellular signaling protein. SdpC strongly upregulates transcription of a two-gene operon, *sdpRI* [74] (previously termed *yvbA* and *yvaZ,* respectively [21]), located immediately downstream from, and in convergent orientation to, the *sdpABC* operon.

Losick and colleagues [21] propose an intriguing model in which a PCD pathway enables a *Bacillus* population to delay sporulation ([21,75]; Figure 4). When nutrients are limited, Spo0A, the key regulatory protein, is activated only in part of the population (the subpopulation of Spo0A-ON cells); in the rest of the cell population, Spo0A remains inactive (the subpopulation of Spo0A-OFF cells). So, according to their model [21], when death by starvation is imminent for the whole population, the following regulatory cascade takes place (Figure 4): i) in Spo0A-ON cells, the *skf* operon is induced, and as a consequence, the cells produce the killing factor mediated by *skf* and the pump (SkfE and SkfF) that exports it, thereby protecting the Spo0A-ON cells from being killed. Because the Spo0A-OFF cells produce neither the killing factor nor the pump, they are killed by the extracellular factor; ii) moreover, in Spo0A-ON cells, the *sdpABC* operon is also induced, leading to the production of SdpC, the toxic signal protein. Cells are self-resistant to SdpC toxin because the *sdpRI* operon, located adjacent and in a convergent orientation to *sdpC* (Figure 4), encodes immunity functions that protect the Spo0A-ON cells from the toxic effect of SdpC (74). The immunity protein SdpI is a putative polytopic membrane protein, and SdpR is an autorepressor that allows only basal expression of the *sdpRI* immunity operon. SdpI is also a signal transduction protein that responds to SdpC by sequestering the SdpR autorepressor at the membrane. Thus SdpC is both a toxin and a ligand; SdpI is both an immunity protein and a receptor/signal transduction protein. Furthermore, in addition to this three-protein intercellular signaling pathway (SdpC–SdpI–SdpR), another control mechanism participates. The repressor AbrB blocks even basal expression of the immunity operon *sdpIR* in Spo0A-OFF cells when these cells are challenged with SdpC toxin/ligand. Conversely, in Spo0A-ON cells, Spo0A represses the gene for AbrB, thereby releasing the *sdpRI* operon from repression [76]. Thus, the immunity operon turns on when the toxin/ligand SdpC is present and the repressor AbrB is absent (Figure 4).

In summary, a cellular differentiation occurs in which Spo0A-ON cells live and Spo0A-OFF cells die due to SdpC protein and the as-yet-unidentified killing factor which causes cell lysis. Their death releases nutrients that are then used by Spo0A-ON cells. Using this “emergency food source,” Spo0A-ON cells can continue growing rather than completing the morphogenic process of spore formation.

As Losick and colleagues suggest [21], such differentiation might be useful for bacterial cell populations, because sporulation is an energy-intensive process that becomes irreversible after its earlier stages. If, during this period, food resources were to become available, sporulating cells would be at a disadvantage compared with cells able to start growing immediately. Thus, for the bacterial population as a whole, delaying the onset of sporulation could be beneficial.

## Conclusions, Questions, and Other “Multicellular” Behaviors

Two different genetic programs were described that promote PCD in bacteria: i) the E. coli toxin–antitoxin module *mazEF*; and ii) the *skf* and *sdp* operons of B. subtilis that mediates the death of some sporulating bacterial cells. In both cases, many intriguing unanswered questions remain. In the case of *E. coli mazEF*: what elements are involved in the death pathway(s)? Do other choromosomally borne toxin–antitoxin systems also participate in such a death process? Do *mazEF*-like systems (Table 1) mediate cell death in the other bacteria? Does *mazEF* mediate cell death in the most devastating pathogens such as *M. tuberculosis, S. aureus,* and B. anthracis ([27,29] and Table 1), and could this death be part of their pathogenicity? The products of *mazEF* [28] and of other bacterial toxin–antitoxin systems [29] might be potential targets for new antibiotics. In particular, it might be possible to design specific antibiotics, the design of which might be greatly facilitated by the already determined crystal structures of MazE [77,78], MazE–MazF [40], and RelB–RelE [79].

The PCD response in *B. subtilis,* which is mediated by *skf* and *sdp* operons, has been studied genetically and physiologically [21,74]. We expect that future studies will reveal information on specific unanswered questions such as, is the killing factor actually a product of the *skf* operon, or is it only mediated by it? Do SkfE and SkfF produce a pump? How does SdpC kill the cells? Is the protein-signaling pathway (SdpC–SdpI–SdpR) sufficient for the immunity response?

Bacterial PCD is a very basic, and at first glance, counterintuitive, phenomenon, making its recent discovery exciting. Future studies of PCD in bacteria will be important for revealing the death pathways involved, and for answering the questions above. The existence of bacterial PCD suggests an important conceptual change in our understanding of bacteria. Growing experimental evidence suggests that bacteria seldom behave as individual organisms. As populations, they manifest “multicellular-like” behaviors, of which PCD may be one. Interesting examples include the ability of bacteria to communicate with each other via quorum-sensing signal molecules [80–90], an important theme in current microbiology. Some basic characteristics of the apparent multicellularity of bacterial populations include differentiation [91–94], intercellular communication [80–90], and PCD [14]. Both the chromosomal toxin–antitoxin PCD systems and the killing response of B. subtilis during sporulation are examples of multicellular behaviors under stressful conditions. When challenged, the bacterial population seems to act like a multicellular organism in which a subpopulation dies, thereby permitting the survival of the bacterial population as a whole. Regarding the role of bacterial PCD in multicellular phenotypes, a main question to be answered is whether, as in *B. subtilis,* cell–cell signaling is involved in *E. coli mazEF*–mediated cell death.

## Abbreviations

PCD: programmed cell death
TLD: thymine-less death
